# Exposure to Road Traffic Noise and Behavioral Problems in 7-Year-Old Children: A Cohort Study

**DOI:** 10.1289/ehp.1409430

**Published:** 2015-06-30

**Authors:** Dorrit Hjortebjerg, Anne Marie Nybo Andersen, Jeppe Schultz Christensen, Matthias Ketzel, Ole Raaschou-Nielsen, Jordi Sunyer, Jordi Julvez, Joan Forns, Mette Sørensen

**Affiliations:** 1 Danish Cancer Society Research Centre, Danish Cancer Society, Copenhagen, Denmark; 2 Section of Social Medicine, Department of Public Health, University of Copenhagen, Copenhagen, Denmark; 3 Department of Environmental Science, Aarhus University, Roskilde, Denmark; 4 Center for Research in Environmental Epidemiology, Barcelona, Spain; 5 Department of Genes and Environment, Norwegian Institute of Public Health, Oslo, Norway

## Abstract

**Background:**

Exposure to traffic noise has been associated with adverse effects on neuropsychological outcomes in children, but findings with regard to behavioral problems are inconsistent.

**Objective:**

We investigated whether residential road traffic noise exposure is associated with behavioral problems in 7-year-old children.

**Methods:**

We identified 46,940 children from the Danish National Birth Cohort with complete information on behavioral problems at 7 years of age and complete address history from conception to 7 years of age. Road traffic noise (L_den_) was modeled at all present and historical addresses. Behavioral problems were assessed by the parent-reported Strengths and Difficulties Questionnaire (SDQ). Associations between pregnancy and childhood exposure to noise and behavioral problems were analyzed by multinomial or logistic regression and adjusted for potential confounders.

**Results:**

A 10-dB increase in average time-weighted road traffic noise exposure from birth to 7 years of age was associated with a 7% increase (95% CI: 1.00, 1.14) in abnormal versus normal total difficulties scores; 5% (95% CI: 1.00, 1.10) and 9% (95% CI: 1.03, 1.18) increases in borderline and abnormal hyperactivity/inattention subscale scores, respectively; and 5% (95% CI: 0.98, 1.14) and 6% (95% CI: 0.99, 1.12) increases in abnormal conduct problem and peer relationship problem subscale scores, respectively. Exposure to road traffic noise during pregnancy was not associated with child behavioral problems at 7 years of age.

**Conclusions:**

Residential road traffic noise in early childhood may be associated with behavioral problems, particularly hyperactivity/inattention symptoms.

**Citation:**

Hjortebjerg D, Andersen AM, Christensen JS, Ketzel M, Raaschou-Nielsen O, Sunyer J, Julvez J, Forns J, Sørensen M. 2016. Exposure to road traffic noise and behavioral problems in 7-year-old children: a cohort study. Environ Health Perspect 124:228–234; http://dx.doi.org/10.1289/ehp.1409430

## Introduction

Exposure to traffic noise is considerable in many parts of the world and has been associated with health effects among adults, including psychological symptoms such as anxiety and changes in mood (Stansfeld and Matheson 2003). Children are also suspected to be vulnerable to traffic noise, especially during sensitive stages of development (Stansfeld et al. 2005). Studies investigating effects on neuropsychological development due to traffic noise exposure in children have focused mainly on learning and cognitive performance, with consistent findings of impairment in reading and memory of aircraft noise exposure (Haines et al. 2001a, 2001b; Hygge et al. 2002; Stansfeld et al. 2005). The few studies that have investigated associations between exposure to traffic noise and parent-reported child behavioral problems are inconsistent (Haines et al. 2001a, 2001b; Stansfeld et al. 2009; Tiesler et al. 2013). Two small studies of schools near Heathrow airport found, respectively, no association and a weak association between school exposure to airport noise and hyperactivity and psychosocial morbidity (Haines et al. 2001a, 2001b). In 2009, a study of > 2,000 children from schools near airports in three European countries found that school exposure to airport noise was associated with an increased score of hyperactivity, whereas exposure to road traffic noise at the schools was not associated with hyperactivity, but with lower scores for conduct problems (i.e. fewer conduct problems) (Stansfeld et al. 2009). The only study investigating associations between residential exposure to road traffic noise and behavioral problems in children reported associations with hyperactivity and possibly emotional symptoms in a study of 900 German children (Tiesler et al. 2013).

Residential exposure to traffic noise might be a more relevant exposure window than exposure at school with regard to the investigated behavioral problems. First, children spend more time at home than at the school; and second, nighttime exposure might be very important, because traffic noise at normal urban levels has been associated with sleep disturbance, with regard to both quality and quantity (Pirrera et al. 2010). In children, sleep disturbance and sleep problems are suspected to affect child behavior (Gregory and Sadeh 2012; Quach et al. 2009), possibly through sleep deficits, which affect the frontal lobe—the part of the brain region that, among other functions, controls behavior and emotions (Quach et al. 2009).

No studies have investigated associations between exposure to traffic noise during pregnancy and behavioral problems. However, noise is an environmental stressor (Stansfeld and Matheson 2003), and maternal exposure to stress during pregnancy has been suggested to be associated with psychological effects in children, including cognitive, behavioral, and emotional development (Graignic-Philippe et al. 2014). A potential mechanism is activation of the maternal hypothalamic–pituitary–adrenal axis, leading to an increase in levels of maternal cortisol (Beijers et al. 2014). Cortisol can pass the fetal–placental barrier and might subsequently influence the fetal nervous system and emotional and cognitive functioning of the child (Davis and Sandman 2012; Seckl and Holmes 2007). Also, maternal sleep disturbance during pregnancy has been proposed to affect the neuroendocrine system (Beijers et al. 2014).

We used data from a large population-based birth cohort to investigate the associations between exposures to road traffic noise at the residence during pregnancy and early life and behavioral problems in 7-year-old children.

## Materials and Methods

*Study population.* The study is based on the population-based Danish National Birth Cohort (DNBC) (Olsen et al. 2001). During 1996–2002, pregnant women who met the inclusion requirements of intending to carry their pregnancy to term, being able to speak Danish, and having a permanent address in Denmark were invited to participate in the DNBC. The invitation took place at the office of the general practitioner, where the women received written information and an informed consent to sign. All participating women provided informed consent.

Participation involved two prenatal computer-assisted telephone interviews conducted by trained interviewers. The first interview took place around the 12th pregnancy week and included, among others, questions related to maternal lifestyle factors during pregnancy, such as alcohol consumption and smoking habits as well as questions related to maternal mental health. Furthermore, when the child was 7 years old, a follow-up questionnaire was mailed to the parents of the child. This 7-year questionnaire included, among others, questions regarding behavioral problems of the child and was based on the Strengths and Difficulties Questionnaire (SDQ).

The DNBC was conducted in accordance with the Helsinki Declaration and approved by the Danish ethics committee.

*Assessment of behavioral problems.* Behavioral problems at 7 years of age were assessed by the Danish parent-reported version of the SDQ (SDQ-Dan) (Goodman 1997; Goodman et al. 2003; Obel et al. 2003). The SDQ is an internationally validated behavioral screening questionnaire for children and adolescents. It encompasses the child’s behavior in the preceding 6 months and is used worldwide for clinical and research purposes.

The SDQ consists of 25 items and generates scores within five subscales: emotional symptoms, conduct problems, hyperactivity/inattention, peer relationship problems, and prosocial behaviors. Each subscale is covered by five items, which can be rated with a three-point scale option: “not true” (0), “somewhat true” (1), or “certainly true” (2), and each subscale score is generated by summing up the ratings. The total difficulties score is obtained by summing up all subscale score except the prosocial behavior score as described in detail elsewhere (Youth*in*Mind 2015). The higher the scores are within each scale, the more behavioral problems are indicated (except for the prosocial behavior score).

In the present study, the total difficulties score and the scores within the subscales of emotional symptoms, conduct problems, hyperactivity/inattention, and peer relationship problems were divided into the categories normal, borderline, or abnormal, by the use of the normative age- and sex-specific cut-off scores for Danish children (Niclasen et al. 2012; Youth*in*Mind 2015). Only children with no missing values on the items were included.

*Exposure.* Residential address history during pregnancy and from birth until 7 years of age was collected using the Danish civil registration system (Pedersen 2011). Road traffic noise exposure was calculated for the years 1995, 2000, 2005, and 2010 for all present and historical addresses using SoundPLAN, which calculates road traffic noise in accordance with the Nordic prediction method (Bendtsen 1999). Based on this, for each child we calculated time-weighted exposures (during pregnancy and childhood), taking all addresses the child had lived in during the period of interest into account, weighted by the time the child had lived at each address.

For each address, the geographical coordinates and height (floor), corresponding to the point of noise estimation were used as input variables for the noise model, including data on road lines, with information on yearly average daily traffic, vehicle distribution (light, heavy), traffic speed, and road type, obtained from DCE-Danish Centre for Environment and Energy, Aarhus University (http://www.dce.au.dk) and from The Danish Road Directorate (http://www.vejdirektoratet.dk), as described in detail elsewhere (Jensen et al. 2009). Topographical parameters included data on building polygons for all surrounding buildings, as well as data on building height, provided by the Danish Geodata Agency (http://www.eng.gst.dk). We assumed that the terrain was flat, which is a reasonable assumption in Denmark, and that urban areas, roads, and areas with water were hard surfaces, whereas all other areas were acoustically porous. No information was available on noise barriers or type of asphalt.

Road traffic noise was calculated as the equivalent continuous A-weighted sound pressure level (L_Aeq_), at the most exposed facade of the dwelling at each address for the day (L_d_; 0700–1900 hours), evening (L_e_; 1900–2200 hours), and night (L_n_; 2200–0700 hours). Road traffic noise was expressed as L_den_ by applying a 5-dB penalty for the evening and a 10-dB penalty for the night. Decibel is a logarithmic scale, which means that a 3-dB higher level of noise corresponds to a doubling in acoustical energy. All values < 40 dB were set to 40 dB because this was considered the lower limit of road traffic noise.

Residential exposure to railway traffic noise was calculated for the years 1995, 2000, 2005, and 2010 for all present and historical addresses using SoundPLAN, which calculates railway traffic noise in accordance with NORD2000, a Nordic calculation method for prediction of noise propagating for railway traffic noise (http://www.soundplan.dk). Geographical coordinates and height (floor) for each residential address were used in the noise model, including railway lines, with information on annual average daily train lengths, train types, and travel speed, which were obtained from the railway enterprise Banedanmark, operating and developing the Danish state railway network (http://www.bane.dk). The daily train lengths were given for 1997 and 2012. Furthermore, building polygons were included in the model as well as all noise barriers along the railway. Railway traffic noise was expressed as L_den_ at the most exposed facade of the dwelling. In the analyses, railway noise exposure < 20 dB was set to 0 because we estimated overall background noise to be no lower than 20 dB.

The noise impact from all Danish airports and airfields was determined from information about noise zones (5-dB categories) obtained from local authorities. The programs DANSIM (Danish Airport Noise Simulation Model) and INM3 (Integrated Noise Model), which fulfill the joint Nordic criteria for air traffic noise calculations were used (Liasjø and Granøien 1993). The curves for airport noise were transformed into digital maps and linked to each residential address history by geographical coordinates.

Air pollution at all geographical coordinates was calculated with the use of the Danish AirGIS modeling system, as described in detail elsewhere (Ketzel et al. 2011). This system allows calculation of air pollution as the sum of local air pollution from traffic in the streets based on the Operational Street Pollution Model (OSPM), the urban background contribution based on an area dispersion model, and contributions from the regional background (Berkowicz et al. 2008). We used levels of nitrogen oxides (NO_x_) as an indicator of air pollution, which was calculated based on data for the relevant years about traffic data for individual road lines, emission factors for Danish car fleet, street and building geometry, including building height as well as meteorological data. Air pollution exposure was expressed as the yearly mean concentration of NO_x_ (micrograms per cubic meter). We focused on NO_x_ as proxy for air pollution from traffic because measured NO_x_ correlates strongly with other traffic-related pollutants in Danish streets including total particle number concentration (10–700 nm; *r* = 0.93) and PM_10_ (particulate matter ≤ 10 μm) (*r* = 0.70) (Ketzel et al. 2003).

*Statistical analyses.* The associations between exposure to residential road traffic and railway noise and behavioral problems at 7 years of age were analyzed by multinomial logistic regression models (for road traffic noise) and logistic regression models (for railway noise). Exposure to road traffic noise was modeled as time-weighted mean during two different exposure windows: *a*) pregnancy period, and *b*) from birth to 7 years of age, taking all present and historical addresses into account. Exposure to railway noise was modeled as continuous at the residential address at *a*) time of birth, and *b*) time of filling in the SDQ (7 years) and was analyzed as a categorical variable among all participants (unexposed, ≤ 60 dB, and > 60 dB) and as a linear trend (per 10 dB) in the subset of the children with railway noise exposure. The assumption of linearity of road traffic and railway noise for both exposure windows in relation to child behavioral problems was evaluated by fitting models with the exposure variables on continuous scale simultaneously with the quadratic term of the exposure variables. All were found to be linear (*p* > 0.05) except for the total difficulties score with regard to road traffic noise exposure from birth until 7 years of age, which was borderline linear (*p* = 0.04).

For road traffic noise, we estimated the associations as odds ratios (OR) with corresponding 95% confidence intervals (CI) for being classified in the borderline or in the abnormal category per 10-dB increase in L_den_ road using the normal category as a reference. For railway noise, we estimated OR for being classified as abnormal using the normal/borderline category as a reference. We calculated crude ORs and adjusted for potential confounders, selected *a priori*, using a two-stage approach. First, models were adjusted for sex, age at filling in the SDQ (years), gestational age (< 37, ≥ 37 weeks), birth weight (< 2,500, ≥ 2,500 g, from the Danish Medical Birth Registry), maternal age at delivery (years), parity (0, 1, ≥ 2), smoking during the first trimester of pregnancy (no/yes), average alcohol consumption (< 1, ≥ 1 drinks per week) during the first trimester of pregnancy, level of education [highest attained education 1 year before conception: basic (7–12 years of primary, secondary, and grammar-school education), vocational (10–12 years of education), and higher (≥ 13 years of education)], disposable income (quintiles; household income after taxation and interest per person, adjusted for number of persons in the household and deflated according to the 2000 value of the Danish crown), railway (no, ≤ 60 dB, > 60 dB) and airport noise (yes, no) at birth (for analyses of road traffic exposure during pregnancy) and at 7 years of age (for analyses of childhood exposure), and maternal mental health problems during the first trimester (“yes” or “no” based on the following two questions in the 12-week pregnancy interview: “Have you ever had psychological disorders or bad nerves?” and “have you had nuisance of this disorder during pregnancy?”). Second, analyses of road traffic noise were further adjusted for time-weighted mean of NO_x_ (micrograms per cubic meter) corresponding to each exposure window. All information on socioeconomic position (SEP)—maternal education and disposable income—was obtained from the national register, Statistics Denmark (http://www.dst.dk). Also, a categorical analysis with five road traffic noise categories of < 50, 50–55, 55–60, 60–65, and ≥ 65 dB was performed for the total difficulties score and the hyperactivity/inattention subscale.

Potential modification of the association between road traffic noise from birth until 7 years of age (per 10-dB increase in road traffic) and the total difficulties score as well as the hyperactivity/inattention subscale by sex, birth weight, educational level, income, and railway noise were evaluated by including interaction terms into a logistic regression model. Potential effect modifiers were selected *a priori* based on previous studies (Lercher et al. 2002). Both scales were dichotomized into abnormal versus normal/borderline behavior, and potential effect modifications were tested by the Wald test. An alpha level of 5% (two-sided) was used to define statistical significance. All analyses were done in SAS (version 9.3; SAS Institute Inc., Cary, NC, USA).

## Results

Of the study base of participating mother–child pairs with information on SDQ (*n* = 57,281), we included only the first enrolled pregnancy to avoid non-independent observations (*n* = 54,103) and excluded 2,272 mothers with multiple pregnancies, 1,833 with incomplete information on behavioral problems, 170 with missing noise exposure data, and 2,888 with incomplete information on one or more potential confounders, leaving a study cohort of 46,940 children.

Characteristics of the study population and cases classified as borderline and abnormal on the total difficulties score are summarized in Table 1. Of the 46,940 children, 11% were classified as borderline and 8% were classified as abnormal. Compared with the cohort, borderline and abnormal cases were more likely to be boys, be the firstborn child, be exposed to maternal smoking during the first trimester, and have mothers with lower educational level and disposable income. The correlation (*R*) between L_den_ road during pregnancy and childhood was 0.74, and between L_den_ road and air pollution (NO_x_) the correlation was 0.59 for the pregnancy period and 0.42 for the period from birth until 7 years of age. There was a high correlation between the L_den_ and L_n_ road for the pregnancy period (0.97) and during childhood (0.90). The correlation between L_den_ road and L_den_ railway among the participants exposed to railway noise (13.1% at 7 years of age) was very weak (0.03).

**Table 1:** Characteristics of the study population by case status using the total difficulties score.

Covariates	Cohort (*n* = 46,940)	Borderline cases^*a*^ (*n* = 5,309)	Abnormal cases^*a*^ (*n* = 3,770)
Sex
Boy	51.1	54.4	52.3
Girl	48.9	45.6	47.7
Age at SDQ (years)	7.13 (7.03–7.41)	7.14 (7.03–7.41)	7.14 (7.03–7.41)
Gestational age at birth (weeks)
< 37	4.2	4.9	6.4
≥ 37	95.8	95.1	93.6
Birth weight (g)
< 2,500	2.6	3.3	4.6
≥ 2,500	97.4	96.7	95.4
Maternal age at birth (years)	30.3 (23.9–37.9)	29.5 (23.1–37.3)	29.1 (22.0–37.3)
Parity
Nulliparous	49.9	56.7	56.0
Uniparous	34.6	31.8	32.7
Multiparous	15.5	11.5	11.3
Maternal smoking during 1st trimester
No	75.9	71.5	65.8
Yes	24.1	28.5	34.2
Maternal alcohol consumption during 1st trimester
< 1 drinks per week	88.2	89.8	90.1
≥ 1 drinks per week	11.8	10.2	9.9
Highest attained education
Basic (7–12 years)	13.3	18.7	26.9
Vocational (10–12 years)	52.8	54.8	53.9
Higher (≥ 13 years)	33.9	26.5	19.2
Disposable income
Low	17.9	18.7	21.3
Medium	30.7	31.4	31.9
High	51.4	49.9	46.8
Maternal mental health problems during 1st trimester
No	98.9	98.5	97.8
Yes	1.1	1.5	2.2
Road traffic noise (dB)^*b*^	57.9 (50.3–68.1)	58.1 (50.5–68.2)	58.6 (50.6–68.1)
Exposed to railway noise at 7 years of age
No	86.9	87.0	86.8
Yes	13.1	13.0	13.2
Among exposed (dB)	48.4 (34.6–64.8)	47.7 (34.8–65.8)	49.6 (34.7–66.0)
Exposed to railway noise at birth
No	84.3	84.3	83.9
Yes	15.7	15.7	16.1
Among exposed (dB)	50.5 (30.6–67.9)	50.4 (30.0–67.9)	51.2 (29.6–68.7)
Exposed to airport noise at 7 years of age	1.3	1.3	1.5
Air pollution (NO_x_, μg/m^3^)^*b*^	12.2 (10.9–34.5)	12.2 (10.9–33.5)	12.3 (10.9–33.3)
Values are percent or median (5th–95th percentiles). ^***a***^Total difficulties score. ^***b***^Mean time-weighted exposure from birth until 7 years of age.			

**Figure 1:**
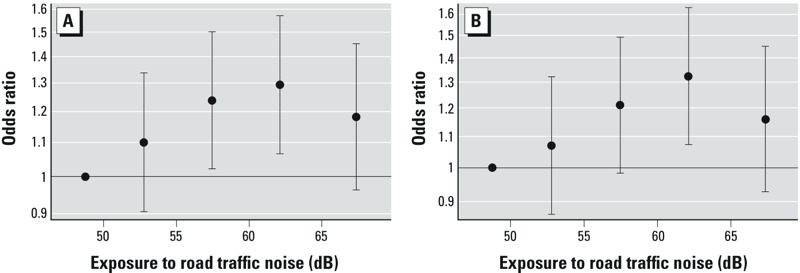
Associations between exposure to road traffic noise (L_den_) at childhood and abnormal scores on the total difficulties score (*A*) and hyperactivity/inattention subscale (*B*). The vertical whiskers show odds ratios with 95% confidence intervals at the median of four exposure categories (50–55, 55–60, 60–65, and ≥ 65 dB) when compared with the reference category of < 50 dB.

For time-weighted mean exposure from birth to 7 years of age, we estimated that a 10-dB higher exposure to road traffic noise was associated with a 7% increase in abnormal total difficulties scores (95% CI: 1.00, 1.14) (Table 2), which seemed to follow a monotonic exposure–response relationship until 60–65 dB, after which the curve leveled off (Figure 1A). On the hyperactivity/inattention subscale, a 10-dB higher road traffic noise exposure was associated with a 5% increase in borderline (95% CI: 1.00, 1.10) and a 10% increase in abnormal (95% CI: 1.03, 1.18) scores as compared with normal scores in the adjusted models (Table 2), which seemed to follow a monotonic exposure–response relationship until 60–65 dB, after which the curve leveled off (Figure 1B). A 10-dB higher exposure to road traffic noise was associated with a 5% increase in abnormal conduct problem scores (95% CI: 0.98, 1.14) and with a 6% increase in abnormal peer relationship scores (95% CI: 0.99, 1.12). Further adjustment for NO_x_ resulted in small increases in the estimates (results not shown). Also, NO_x_ exposure in itself (in models without adjustment for noise) was not associated with behavioral problems: For example, a 20-μg/m^3^ increase in time-weighted mean exposure to NO_x_ from birth to 7 years was associated with ORs of 0.95 (95% CI: 0.90, 1.00) and 0.95 (95% CI: 0.89, 1.01) for scoring borderline and abnormal, respectively, on the total difficulties score, and of 0.97 (95% CI: 0.92, 1.02) and 0.99 (95% CI: 0.93, 1.06) for scoring borderline and abnormal, respectively, on the hyperactivity/inattention subscale. There were no clear associations between exposure to road traffic noise during pregnancy and behavioral problems (Table 2). Exposure during pregnancy was inversely associated with borderline total difficulties scores (OR = 0.95; 95% CI: 0.90, 0.99) but was not associated with abnormal total difficulties scores (OR = 0.99; 95% CI: 0.94, 1.05). For both exposure time windows, adjusting for airport and railway noise did not affect associations of road traffic noise with borderline or abnormal scores for total difficulties score or any of the subscales (see Supplemental Material, Table S1). Adjusting for road traffic and airport noise had no influence on odds ratios for railway noise at birth or at 7 years of age (see Supplemental Material, Table S2).

**Table 2:** Associations between exposure to road traffic noise (L_den_, per 10-dB increase) during pregnancy and early childhood and child behavioral borderline or abnormal scores.

Strengths and Difficulties Questionnaire (SDQ)	Exposure to road traffic noise (L_den_) during pregnancy^*a*^			Exposure to road traffic noise (L_den_) from birth to 7 years of age^*a*^		
	*n*	Crude OR (95% CI)	Adjusted OR (95% CI)^*b*^	*n*	Crude OR (95% CI)	Adjusted OR (95% CI)^*b*^
Total difficulties
Normal	37,861	1.00	1.00	37,861	1.00	1.00
Borderline	5,309	0.99 (0.95, 1.04)	0.95 (0.90, 0.99)	5,309	1.07 (1.01, 1.13)	1.00 (0.95, 1.06)
Abnormal	3,770	1.05 (1.00, 1.11)	0.99 (0.94, 1.05)	3,770	1.17 (1.11, 1.25)	1.07 (1.00, 1.14)
Emotional symptoms
Normal	40,245	1.00	1.00	40,245	1.00	1.00
Borderline	3,099	1.08 (1.02, 1.15)	1.00 (0.95, 1.06)	3,099	1.12 (1.05, 1.19)	1.03 (0.96, 1.10)
Abnormal	3,596	1.08 (1.02, 1.14)	0.97 (0.92, 1.03)	3,596	1.11 (1.04, 1.18)	0.98 (0.92, 1.05)
Conduct problems
Normal	40,374	1.00	1.00	40,374	1.00	1.00
Borderline	4,045	0.99 (0.94, 1.04)	0.99 (0.94, 1.05)	4,045	1.02 (0.97, 1.09)	1.01 (0.96, 1.07)
Abnormal	2,521	0.98 (0.92, 1.05)	0.98 (0.92, 1.05)	2,521	1.10 (1.03, 1.18)	1.05 (0.98, 1.14)
Hyperactivity/inattention
Normal	37,799	1.00	1.00	37,799	1.00	1.00
Borderline	6,097	1.03 (0.99, 1.08)	1.01 (0.96, 1.05)	6,097	1.09 (1.05, 1.15)	1.05 (1.00, 1.10)
Abnormal	3,044	1.04 (0.99, 1.11)	1.01 (0.96, 1.08)	3,044	1.18 (1.11, 1.26)	1.10 (1.03, 1.18)
Peer relationship problems
Normal	37,690	1.00	1.00	37,690	1.00	1.00
Borderline	5,243	1.02 (0.98, 1.07)	1.01 (0.97, 1.06)	5,243	1.06 (1.01, 1.12)	1.05 (0.99, 1.10)
Abnormal	4,007	1.02 (0.97, 1.08)	0.99 (0.94, 1.04)	4,007	1.12 (1.06, 1.19)	1.06 (0.99, 1.12)
^***a***^Mean time-weighted exposure. ^***b***^Adjusted for sex, age at SDQ, gestational age, birth weight, maternal age at delivery, parity, educational level, disposable income, smoking and alcohol consumption during 1st trimester, railway and airport noise at birth (for exposure during pregnancy) and at 7 years of age, and self-reported maternal mental health problems during 1st trimester (yes/no).						

Among the subset of children with railway noise exposure at 7 years, a 10-dB increase in exposure was positively associated with abnormal scores for total difficulties (OR = 1.13; 95% CI: 1.02, 1.25) and peer relationship problems (OR = 1.13; 95% CI: 1.03, 1.25) (Table 3). We found no significant associations between this exposure and the remaining outcomes, though for the hyperactivity/inattention subscale a 10-dB increase in railway noise was associated with a 9% increase in abnormal scores (95% CI: 0.97, 1.22). In the cohort as a whole, exposure to railway noise ≤ 60 dB at the time of birth was positively associated with abnormal emotional symptom scores (OR = 1.11; 95% CI: 1.00, 1.23 compared with unexposed children) but this outcome was not associated with railway noise > 60 dB (OR = 1.01; 95% CI: 0.83, 1.22). No other associations between exposure to railway noise at the time of birth and abnormal behavioral problems were observed.

**Table 3:** Associations between exposure to railway noise at time of birth and at SDQ (7 years), and abnormal scores on the total difficulties score and sub scales.

Strengths and Difficulties Questionnaire (SDQ)	Exposure to railway noise (L_den_) at time of birth			Exposure to railway noise (L_den_) 7-year SDQ		
	Abnormal cases (*n*)	Crude OR (95% CI)	Adjusted OR (95% CI)^*a*^	Abnormal cases (*n*)	Crude OR (95% CI)	Adjusted OR (95% CI)^*a*^
Total difficulties score (*n* = 3,770)
Unexposed	3,164	1.00	1.00	3,270	1.00	1.00
≤ 60 dB	473	0.98‑(0.88,‑1.08)	0.98‑(0.88,‑1.08)	420	0.95‑(0.96,‑1.06)	0.94‑(0.85,‑1.06)
> 60 dB	133	1.04 (0.87, 1.25)	0.97 (0.81, 1.17)	80	1.20 (0.95, 1.52)	1.14 (0.90, 1.45)
Linear trend per 10 dB^*b*^	606	1.03 (0.89, 1.12)	1.01 (0.93, 1.10)	500	1.15 (1.04, 1.27)	1.13 (1.02, 1.25)
Emotional symptoms (*n* = 3,596)
Unexposed	2,957	1.00	1.00	3,085	1.00	1.00
≤ 60 dB	509	1.14 (1.03, 1.26)	1.11 (1.00, 1.23)	439	1.17 (0.96, 1.19)	1.05 (0.94, 1.16)
> 60 dB	130	1.09 (0.91, 1.31)	1.01 (0.83, 1.22)	72	1.14 (0.89, 1.45)	1.10 (0.86, 1.41)
Linear trend per 10 dB^*b*^	639	1.03 (0.95, 1.12)	1.02 (0.94, 1.11)	511	1.01 (0.91, 1.12)	1.00 (0.90, 1.11)
Conduct problems (*n* = 2,521)
Unexposed	2,128	1.00	1.00	2,174	1.00	1.00
≤ 60 dB	313	0.96 (0.86, 1.09)	0.98 (0.87, 1.11)	300	1.03 (0.91, 1.17)	1.05 (0.92, 1.18)
> 60 dB	80	0.92 (0.73, 1.16)	0.90 (0.71, 1.13)	47	1.04 (0.77, 1.41)	1.01 (0.75, 1.37)
Linear trend per 10 dB^*b*^	393	0.96 (0.87, 1.06)	0.94 (0.85, 1.04)	347	0.96 (0.85, 1.04)	0.95 (0.84, 1.07)
Hyperactivity/inattention (*n* = 3,044)
Unexposed	2,570	1.00	1.00	2,643	1.00	1.00
≤ 60 dB	368	0.94 (0.84, 1.05)	0.94 (0.86, 1.05)	341	0.96 (0.85, 1.08)	0.94 (0.85, 1.07)
> 60 dB	106	1.02 (0.83, 1.25)	0.97 (0.79, 1.19)	60	1.10 (0.85, 1.44)	1.05 (0.80, 1.38)
Linear trend per 10 dB^*b*^	474	0.99 (0.90, 1.09)	0.98 (0.87, 1.07)	401	1.11 (0.99, 1.24)	1.09 (0.97, 1.22)
Peer relationship problems (*n* = 4,007)
Unexposed	3,362	1.00	1.00	3,470	1.00	1.00
≤ 60 dB	509	0.99 (0.90, 1.09)	0.98 (0.89, 1.09)	446	0.96 (0.86, 1.06)	0.96 (0.86, 1.06)
> 60 dB	136	0.99 (0.83, 1.19)	0.97 (0.80, 1.16)	91	1.30 (1.04, 1.62)	1.27 (1.01, 1.58)
Linear trend per 10 dB^*b*^	645	0.98 (0.91, 1.07)	0.98 (0.90, 1.06)	537	1.15 (1.04, 1.27)	1.13 (1.03, 1.25)
^***a***^Adjusted for sex, age at SDQ, gestational age, birth weight, maternal age at delivery, parity, educational level, disposable income, smoking and alcohol consumption during 1st trimester, airport noise at birth (for exposure at birth) and at SDQ, road traffic noise during pregnancy (for exposure at birth) and from birth until 7 years of age, and self-reported maternal mental ill health during 1st trimester (yes/no). ^***b***^Linear association among exposed.						

We found no significant effect modification by sex, low birth weight, educational level, income, or railway noise, though for birth weight we found a borderline significant effect modification (*p* = 0.06), with stronger association between road traffic noise and hyperactivity/inattention for children with low birth weight (Table 4).

**Table 4:** Modification of associations between time-weighted mean exposure to road traffic noise (L_den_) from birth to 7 years of age (per 10-dB increase)*^a^* and abnormal scores on the total difficulties score and the hyperactivity/inattention subscale by sex, birth weight, education, income, and railway noise.

Characteristic	Total difficulties score			Hyperactivity/inattention		
	Abnormal cases (*n*)	OR (95% CI)^*b*^	*p*‑Interaction	Abnormal cases (*n*)	OR (95% CI)^*b*^	*p*‑Interaction
Sex			0.49			0.10
Girl	1,798	1.09 (1.00, 1.19)		1,339	1.16 (1.05, 1.29)
Boy	1,972	1.05 (0.97, 1.14)		1,705	1.04 (0.96, 1.14)	
Birth weight (g)			0.17			0.06
< 2,500	176	1.30 (0.97, 1.76)		140	1.49 (1.08, 2.05)
≥ 2,500	3,596	1.06 (0.99, 1.13)		2,904	1.08 (1.01, 1.16)
Parental educational level			0.98			0.31
Basic	1,016	1.07 (0.94, 1.22)		718	1.11 (0.97, 1.28)
Vocational	2,030	1.06 (0.98, 1.15)		1,653	1.05 (0.96, 1.15)
Higher	724	1.07 (0.94, 1.23)		673	1.19 (1.04, 1.36)
Disposable income^*c*^			0.99			0.72
Low	1,352	1.07 (0.97, 1.19)		1,043	1.12 (1.00, 1.25)
High	2,418	1.07 (0.99, 1.15)		2,001	1.09 (1.01, 1.18)
Railway noise			0.67			0.23
Unexposed	3,270	1.06 (0.99, 1.13)		2,643	1.07 (1.00, 1.15)
≤ 60 dB	420	1.13 (0.95, 1.35)		341	1.28 (1.06, 1.55)
≥ 60 dB	80	0.95 (0.62, 1.45)		60	1.01 (0.63, 1.63)
^***a***^Mean time-weighted exposure. ^***b***^Adjusted for sex, age at SDQ, gestational age, birth weight, maternal age at delivery, parity, educational level, disposable income, smoking and alcohol consumption during 1st trimester, railway and airport noise at 7 years of age, and self-reported maternal mental health problems during 1st trimester (yes/no). ^***c***^Cut point is median income of the Danish background population (age standardized), obtained from Statistics Denmark.						

## Discussion

Using a large national birth cohort study, we found that cumulative exposure during childhood to road traffic noise at home was positively associated with behavioral problems at 7 years of age, particularly hyperactivity/inattention symptoms. We found no consistent associations between exposure to either road traffic or railway noise at home during the pregnancy period and behavioral problems.

Our findings suggest that exposure to residential road traffic noise during childhood and potentially railway noise may increase the risk for hyperactivity/inattention symptoms at 7 years of age. Hyperactive children are normally more easily distracted by background noise (Gray et al. 2002), and it seems possible that traffic noise may exacerbate these children’s difficulties, thereby making an existing tendency toward hyperactivity worse or more obvious. Our results are in line with those of most previous studies investigating associations between exposure to traffic noise either at home and in schools and behavioral problems (Haines et al. 2001b; Stansfeld et al. 2009; Tiesler et al. 2013). A similar though smaller German study (900 children) reported road traffic noise at home (for the address at time of SDQ) to be significantly associated with more hyperactivity/inattention symptoms in 10-year-old children (Tiesler et al. 2013). The published studies on airport and road traffic noise at schools are less consistent. Two studies reported positive associations between road traffic noise and hyperactivity/inattention symptoms (Haines et al. 2001a; Stansfeld et al. 2009), whereas the third study reported no association (Haines et al. 2001b). A possible explanation for this inconsistency might be that exposure to traffic noise at home is potentially more hazardous than school exposure, perhaps because children typically spend more time at home than at school, and that nighttime exposure to noise might be particularly hazardous because it disturbs sleep (Basner et al. 2011; Hume et al. 2012; Pirrera et al. 2010), which is suspected of affecting child behavior (Gregory and Sadeh 2012; Quach et al. 2009). However, we had no information on sleep disturbance among the children and could not separate the effects of nighttime exposure to road traffic noise from daytime exposure because of the high correlation between L_den_ and L_n_; therefore, speculations regarding hazardous effects of nighttime noise in the present study are hypothetical.

One potentially important confounder in the present study is exposure to air pollution, because air pollution is correlated with road traffic noise and is also suspected of having damaging impact on the central nervous system (Block et al. 2012), possibly affecting the cognitive development of children (Guxens et al. 2014). However, NO_x_, an indicator of traffic-related air pollution, was not associated with behavioral problems, and adjustment for it resulted in only minor changes in estimates.

To our knowledge, this is the first study to report a positive association between traffic noise—both road traffic and railway noise—and scoring abnormal on the total difficulties score, corresponding to an estimate of overall behavioral problems. None of the four previous studies investigating this for exposures to traffic noise at home or in school have found traffic noise associated with this score (Haines et al. 2001a, 2001b; Stansfeld et al. 2009; Tiesler et al. 2013). However, these previous studies are smaller than the present study (< 2,014 children), with less power to detect the rather small associations seen in the present study. The total difficulties score is a combination of four behavioral domains, and it seems likely that in our study the association with this score is driven mainly by the positive association found for the hyperactivity/inattention subscale.

We found no associations between road traffic noise and emotional symptoms, and weak, insignificant associations with conduct problem and peer relationship problems. These results are similar to those of studies on school exposure to traffic noise but in contrast with the study by Tiesler et al. on residential road traffic noise, which indicated an association with emotional symptoms (Tiesler et al. 2013). A possible explanation for the different results might be differences in adjustment for potential confounders, because we found positive associations with borderline and abnormal emotional symptom scores in our crude analysis. However, no associations were observed in the adjusted analyses.

Our study indicated that railway noise exposure at 7 years of age was positively associated with peer relationship problems in our study population. However, we have no explanation for this finding; it may be a chance finding, because we find no associations with road traffic noise.

Our results indicated that exposure during pregnancy was not associated with childhood behavior at 7 years of age. The only significant finding was an inverse association for scoring borderline on the total difficulties score, which we believe to be a chance finding because this is opposite our hypothesis and found only for the borderline score and not for the abnormal score. Associations between pregnancy exposure to traffic noise and behavioral problems in childhood have to our knowledge not been investigated before, but our results suggest that prenatal stress due to traffic noise is not important in relation to this outcome.

We found a borderline significant effect modification by birth weight, with stronger association between road traffic noise and hyperactivity/inattention for children with low birth weight. Previous studies on this are inconsistent. One study found that the association between ambient neighborhood noise (predominantly road and railway noise) and mental health problems in children was modified by low birth weight or being born premature, with strongest association among children with low birth weight or prematurity (Lercher et al. 2002). On the other hand, a recent study found no effect modification (interaction *p*-values > 0.05) by low birth weight or preterm birth in relation to the association between school exposure to airport or road traffic noise and children’s mental health (Crombie et al. 2011). More studies in this area are needed.

Strengths of our study include the large study population, with information on various potential confounders obtained from questionnaires and nationwide registers, as well as modeled air pollution. Another major strength is access to residential address histories from conception to 7 years of age, which makes it possible to investigate different exposure time windows.

Some limitations have to be considered. We used the Nordic prediction method for noise estimation, and although the Nordic prediction method has been used for many years, estimation of noise may be associated with some degree of uncertainty. Noise estimation depends on accurate input data, and we had no information on noise barriers or road surface in the modeling of road traffic noise. This could have resulted in exposure misclassification, but such misclassification is believed to be nondifferential, and, in most situations, this would influence the estimates toward the neutral value. In addition, because the correlation between L_den_ and L_n_ was very high in the present study, we were not able to separate the effect of these two exposures. Another limitation is that we had information only on residential addresses of the child and not, for example, the address of a single parent, if the parents were divorced or living apart, with whom the child could be staying part of the time. Moreover, we had no information on whether the child’s bedroom faced a busy road or backyard, or on noise insulation or window-opening habits, all of which influence the child’s personal exposure to noise. Studies have found associations between noise and cardiovascular outcomes to be stronger when factors like these are considered (Foraster et al. 2014; Selander et al. 2009). Therefore, lack of this information might have contributed to underestimation of the effects of road traffic noise and railway noise on behavioral problems. Furthermore, behavioral problems were based on the parent-reported version of the SDQ and recalling of the child’s behavior in the past 6 months, which may be associated with some recall bias. Also, the parental version of the SDQ has in community samples been suggested not to capture emotional symptoms as well as the other subscales, which may have affected the results of this subscale (Goodman et al. 2003). Moreover, maternal mental health problems were based on a combination of two items in the 12th-week pregnancy interview and may not have adequately captured maternal psychopathology. Reviewing medical records to obtain information on confirmed diagnoses and using a time interval longer than the first trimester might have improved the adjustment of this confounder. Last, there might be residual confounding by SEP. However, we have detailed information from questionnaires and registers on the most important confounders. Also, in Denmark a high proportion of highly educated people live in central urban areas with relatively high traffic noise, so differences in noise exposure according to SEP is not pronounced in the present study: Mothers with low, medium, and high levels of education were exposed to medians of 58.8, 57.9, and 57.7 dB road traffic noise, respectively, suggesting that residual confounding by SEP is not a major problem in the present study.

In conclusion, this study provides further insight into the relationship between traffic noise and behavior in children. The results indicate that, in our study population, exposure to residential road traffic noise from birth until 7 years of age was associated with parentally reported hyperactivity/inattention symptoms at 7 years, whereas exposure to noise during pregnancy was not associated with behavioral problems in childhood. More studies are needed to understand the mechanism through which traffic noise might affect children’s behavior.

## Supplemental Material

(105 KB) PDF
