# Supplementary material for: Exposure to Road Traffic Noise and Behavioral Problems in 7-Year-Old Children: A Cohort Study
Source: Environ Health Perspect. 2015 Jun 30;124(2):228–34. doi: 10.1289/ehp.1409430 (PMC4749080; doi:10.1289/ehp.1409430)
Supplement: (105 KB) PDF [file ehp.1409430.s001.acco.pdf]

Environ Health Perspect
DOI: 10.1289/ehp.1409430

Note to Readers: *EHP* strives to ensure that all journal content is accessible to all readers. However, some figures and Supplemental Material published in *EHP* articles may not conform to 508 standards due to the complexity of the information being presented. If you need assistance accessing journal content, please contact ehp508@niehs.nih.gov. Our staff will work with you to assess and meet your accessibility needs within 3 working days.

Supplemental Material

Exposure to Road Traffic Noise and Behavioral Problems in 7-Year-Old Children: A Cohort Study

Dorrit Hjortebjerg, Anne Marie Nybo Andersen, Jeppe Schultz Christensen, Matthias Ketzel, Ole Raaschou-Nielsen, Jordi Sunyer, Jordi Julvez, Joan Forns, and Mette Sørensen

Table of Contents

Table S1. Associations between exposure to road traffic noise (L_{den}, per 10 dB increase) during pregnancy and early childhood and behavioral borderline or abnormal scores without adjustment for railway and airport noise

Table S2. Associations between exposure to railway noise (L_{den}, per 10 dB increase) among exposed during pregnancy and early childhood and abnormal scores without adjustment for road traffic and airport noise

Table S1. Associations between exposure to road traffic noise (L_{den}, per 10 dB increase) during pregnancy and early childhood and behavioral borderline or abnormal scores without adjustment for railway and airport noise

Strengths and difficulties score	Exposure to road traffic noise (L_{den}) during pregnancy [a]<br>Adjusted OR (95% CI) [b]	Exposure to road traffic noise (L_{den}) from birth to 7 years of age [a]<br>Adjusted OR (95% CI) [b]
**Total difficulties score**		
Normal	1.00	1.00
Borderline	0.95 (0.90, 0.99)	1.00 (0.95, 1.06)
Abnormal	0.99 (0.94, 1.05)	1.07 (1.01, 1.14)
**Emotional symptoms**		
Normal	1.00	1.00
Borderline	1.00 (0.95, 1.06)	1.03 (0.97, 1.10)
Abnormal	0.98 (0.92, 1.03)	0.99 (0.93, 1.05)
**Conduct problems**		
Normal	1.00	1.00
Borderline	0.99 (0.94, 1.05)	1.01 (0.95, 1.07)
Abnormal	0.98 (0.92, 1.05)	1.06 (0.98, 1.14)
**Hyperactivity/inattention**		
Normal	1.00	1.00
Borderline	1.01 (0.96, 1.05)	1.05 (1.00, 1.11)
Abnormal	1.01 (0.96, 1.08)	1.11 (1.04, 1.18)
**Peer relationship problems**		
Normal	1.00	1.00
Borderline	1.02 (0.97, 1.06)	1.05 (0.99, 1.10)
Abnormal	0.99 (0.94, 1.04)	1.06 (0.99, 1.12)

[a]Mean time-weighted exposure. [b]Adjusted for sex, age at SDQ, gestational age, birth weight, maternal age at delivery, parity, educational level, disposable income, smoking and alcohol consumption during 1st trimester and self-reported mental health problems during 1st trimester (yes/no)

Table S2. Associations between exposure to railway noise (L_{den}, per 10 dB increase) among exposed during pregnancy and early childhood and abnormal scores without adjustment for road traffic and airport noise

Strengths and difficulties score	Exposure to railway noise (L_{den}) at time of birth<br>Adjusted OR (95% CI) [a]	Exposure to railway noise (L_{den}) at the age of 7 years SDQ<br>Adjusted OR (95% CI) [a]
**Total difficulties score**		
Normal or borderline	1.00	1.00
Abnormal	1.01 (0.93, 1.10)	1.13 (1.02, 1.26)
**Emotional symptoms**		
Normal or borderline	1.00	1.00
Abnormal	1.02 (0.94, 1.10)	1.00 (0.90, 1.11)
**Conduct problems**		
Normal or borderline	1.00	1.00
Abnormal	0.94 (0.85, 1.04)	0.95 (0.84, 1.07)
**Hyperactivity/inattention**		
Normal or borderline	1.00	1.00
Abnormal	0.98 (0.89, 1.07)	1.09 (0.97, 1.22)
**Peer relationship problems**		
Normal or borderline	1.00	1.00
Abnormal	0.98 (0.90, 1.06)	1.14 (1.03, 1.26)

[a] Adjusted for sex, age at SDQ, gestational age, birth weight, maternal age at delivery, parity, educational level, disposable income, smoking and alcohol consumption during 1st trimester and self-reported mental health problems during 1st trimester (yes/no)
